# Foliar Application of Selenium in Mitigating Salinity Stress on the Physiology, Growth, and Yield of Okra

**DOI:** 10.3390/plants15010021

**Published:** 2025-12-20

**Authors:** Allesson Ramos de Souza, Carlos Alberto Vieira de Azevedo, Lucyelly Dâmela Araújo Borborema, Geovani Soares de Lima, Lauriane Almeida dos Anjos Soares, André Alisson Rodrigues da Silva, Kheila Gomes Nunes, Denis Soares Costa, Pedro Henrique Duarte Durval, Thiago Filipe de Lima Arruda, Rosany Duarte Sales, Pâmela Monique Valões da Cruz, Brendo Júnior Pereira Farias, Hans Raj Gheyi, Vera Lúcia Antunes de Lima, Jailton Garcia Ramos

**Affiliations:** 1 Academic Unit of Agricultural Engineering, Federal University of Campina Grande, Campina Grande 58430-380, Brazil; carlos.vieira@professor.ufcg.edu.br (C.A.V.d.A.); lucyellyd@gmail.com (L.D.A.B.); kheilagomesnunes@gmail.com (K.G.N.); denis_soares11@hotmail.com (D.S.C.); pedro2durval2@gmail.com (P.H.D.D.); thiago.filipe@estudante.ufcg.edu.br (T.F.d.L.A.); rdrosany@gmail.com (R.D.S.); valoespamela@hotmail.com (P.M.V.d.C.); brendojr88@gmail.com (B.J.P.F.); hans.gheyi@ufcg.edu.br (H.R.G.); vera.lucia@professor.ufcg.edu.br (V.L.A.d.L.); jgramos2019@gmail.com (J.G.R.); 2 Academic Unit of Agrarian Sciences, Federal University of Campina Grande, Pombal 58840-000, Brazil; geovani.soares@professor.ufcg.edu.br (G.S.d.L.); lauriane.soares@professor.ufcg.edu.br (L.A.d.A.S.); 3 Academic Unit of Agronomy, Universidade Federal do Oeste do Pará, Juruti 68170-000, Brazil; andrealisson_cgpb@hotmail.com

**Keywords:** salinity, beneficial element, *Abelmoschus esculentus*

## Abstract

This study aimed to evaluate the effect of selenium concentrations in mitigating salt stress on the physiology, growth, and yield of okra plants irrigated with brackish water. Treatments consisted of four irrigation water salinity levels (ECw: 0.4, 1.3, 2.2, and 3.1 dS m^−1^) combined with four selenium concentrations (0, 5, 10, and 15 mg L^−1^), arranged in a randomized block design in a 4 × 4 factorial scheme, with three replicates and one plant per plot. Increasing irrigation water salinity from 0.4 dS m^−1^ reduced relative water content, gas exchange, initial chlorophyll *a* fluorescence, plant growth, and production of okra, while increasing the percentage of electrolyte leakage. Irrigation Water salinity levels above 0.4 dS m^−1^ impaired plant water status, gas exchange, growth, chlorophyll a fluorescence, yield, and water-use efficiency, while increasing electrolyte leakage. Salinity above 1.0 dS m^−1^ also inhibited photosynthetic pigment synthesis. Selenium did not mitigate salinity-induced reductions in chlorophyll and carotenoids. However, foliar Se at 8.6–15 mg L^−1^ enhanced gas exchange, chlorophyll *a* fluorescence, growth, and fruit yield under salinity up to 3.1 dS m^−1^. These results support Se induced attenuation of salinity stress, warranting further mechanistic studies.

## 1. Introduction

Okra [*Abelmoschus esculentus* (L.)] is a vegetable that stands out for its nutritional value [1], characterized by expressive amounts of carbohydrates (7 g), proteins (2 g), and dietary fiber (3.2 g) per 100 g of fresh fruit. It also contains a wide range of minerals, such as potassium, calcium, phosphorus, manganese, and several vitamins [2].

In 2017, Brazil recorded a production of 111,967 tons of okra, of which 28.74% (32,186 tons) originated from the semiarid region of Northeastern Brazil. The state of Paraíba produced less than 1.00% of the national output, thus demonstrating low representativeness [3]. This limitation is directly associated with the edaphoclimatic conditions of the region, characterized by the irregular distribution of rainfall in both time and space, high evapotranspiration rates, and the presence of young soils that are often rich in soluble salts and have low water-holding capacity factors that pose significant challenges to agricultural productivity [4].

Salinity in irrigation water and/or soil is one of the main constraints to agricultural production, as it leads to the excessive accumulation of sodium (Na^+^) and chloride (Cl^−^) ions in plant tissues, triggering ionic effects, reducing the uptake of nutrients such as potassium (K^+^) and calcium (Ca^2+^), and impairing metabolic and enzymatic functions, which in turn results in the excessive production of reactive oxygen species (ROS) [5]. These effects compromise cellular homeostasis, inducing oxidative stress and causing damage to cell membranes [6], chloroplasts, and essential physiological functions, including protein synthesis and photosynthesis [7]. In okra, the impact of salinity is mainly expressed through reductions in cellular turgor pressure, disruptions in ionic homeostasis, and decreases in photosynthetic efficiency, all of which ultimately reduce its productive potential [8].

Thus, understanding and developing strategies to alleviate the effects of salt stress in plants is one of the major challenges of modern agriculture. In this regard, the application of compounds with the potential to act as signaling molecules and/or osmoprotectants has gained prominence [7]. Selenium (Se), the 67th most abundant trace element in the Earth’s crust [9], plays an important role in this process by strengthening the antioxidant system, enhancing photosynthesis and carbon secondary metabolism, thereby contributing to increased tolerance to abiotic stresses [10]. This response is mainly associated with the enhanced activity of antioxidant enzymes, particularly the increase in GPX, which reduces hydrogen peroxide (H_2_O_2_) to water and toxic lipid hydroperoxides to their corresponding alcohols, using reduced glutathione as an electron donor [11]. Studies have reported the beneficial effects of selenium on the physiology of common bean (*Phaseolus vulgaris* L.) [12], on the growth of tomato (*Solanum lycopersicum*) [13], and cucumber (*Cucumis sativus*) [14] under salt stress. However, there are no reports on the role of selenium application in the morphophysiology and yield of okra plants irrigated with brackish water.

In this context, selenium stands out as a promising alternative for inducing plant to-lerance to salt stress; however, its excessive use may result in phytotoxicity and relevant environmental impacts, highlighting the need for research aimed at defining appropriate doses and evaluating its effects on plant physiology, growth, and yield [15]. Selenium (Se), when applied at low concentrations has shown to reduce the negative effects of salt stress [16]. Similar results were reported by Amerian et al. [17], in which the application of 10 mg L^−1^ of Se promoted beneficial effects on the morphophysiology of cucumber plants irrigated with brackish water.

The semiarid region of Northeastern Brazil presents several problems associated with the excessive salt content found in many of its soils and/or water sources. Thus, this study was based on the hypothesis that foliar application of selenium mitigates the deleterious effects of salt stress on the physiology, growth, and yield of okra by enhancing enzymatic and antioxidant activity. Therefore, the objective of this study was to evaluate the effect of selenium application on the physiology, growth, and production of okra cultivated in a semiarid area of Northeastern Brazil.

## 2. Results

There was a significant effect (*p* ≤ 0.05) of the interaction between electrical conductivity levels and selenium concentrations (ECw × Se) on the relative water content and electrolyte leakage of the leaf blade (Table S1).

**Figure 1:**
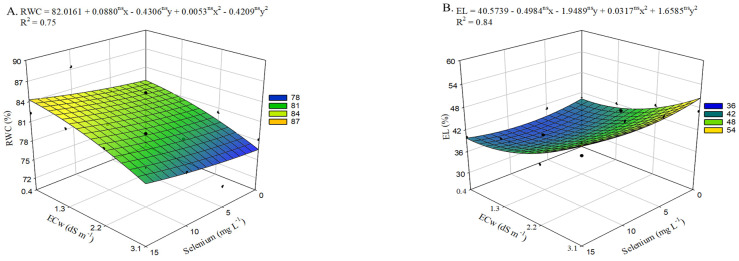
Relative water content—RWC (**A**) and electrolyte leakage—EL (**B**) of okra plants as a function of the interaction between irrigation water electrical conductivity levels and selenium concentrations, at 65 days after sowing. X and Y—Selenium concentrations and irrigation water electrical conductivity, ns—Not significant by F test. Vertical bars represent the standard error of the mean (*n* = 3).

With increasing salinity, a 5.14% reduction in relative water content (Figure 1A) was observed in okra plants when comparing those cultivated with an ECw of 0.4 dS m^−1^ to those irrigated with 3.1 dS m^−1^. The application of selenium at a concentration of 15 mg L^−1^ promoted increases in RWC. However, the highest estimated value, 84.28%, was re-corded in plants irrigated with an ECw of 0.4 dS m^−1^, representing a 2.50% increase compared with plants subjected to the same salinity level but without selenium application (0 mg L^−1^). The beneficial effect of applying selenium at 15 mg L^−1^ was also observed in plants grown under an ECw of 3.1 dS m^−1^, resulting in a 2.51% increase compared with those irrigated at the same conductivity level but not receiving foliar selenium application.

Irrigation with an ECw of 3.1 dS m^−1^ increased electrolyte leakage in the leaf blade (Figure 1B) by 10.41% compared with plants irrigated with the lowest salinity level. On the other hand, selenium concentrations up to 8 mg L^−1^ were able to mitigate the deleterious effects of salt stress, resulting in a reduction in this variable, with the lowest value (38.06%) observed under an ECw of 0.7 dS m^−1^. However, increasing Se concentrations above 8 mg L^−1^ led to an increase in EL, resulting in the highest observed value (50.13%) when plants were subjected to 15 mg L^−1^ and irrigated with an ECw of 3.1 dS m^−1^.

The interaction between electrical conductivity levels (ECw) and selenium concentrations (Se) significantly affected stomatal conductance (*gs*), transpiration (*E*), CO_2_ assi-milation rate (*A*), instantaneous water-use efficiency (*WUEi*), and instantaneous carboxylation efficiency (*CEi*) (Table S2).

**Figure 2:**
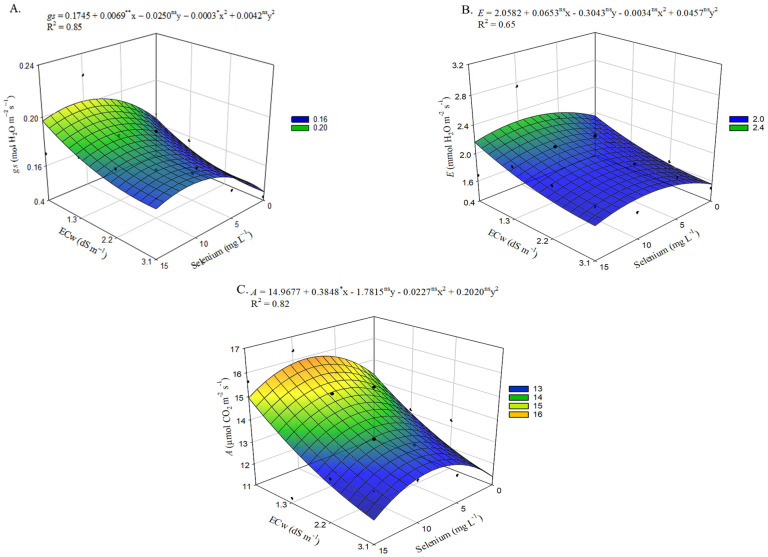
Stomatal conductance—*gs* (**A**), transpiration—*E* (**B**) and CO_2_ assimilation rate—*A* (**C**) of okra plants, as a function of the interaction between the electrical conductivity levels of irrigation water and different concentrations of selenium, at 65 days after sowing. X and Y—Selenium concentrations and irrigation water electrical conductivity, respectively. *, **, and ns—Significant at *p* ≤ 0.05, *p* ≤ 0.01, and not significant by F test, respectively. Vertical bars represent the standard error of the mean (*n* = 3).

Increasing irrigation water electrical conductivity from 0.4 dS m^−1^ to 3.1 dS m^−1^ resulted in a 16.84% reduction in stomatal conductance in okra plants (Figure 2A). However, the application of selenium (Se) at estimated concentration of 11 mg L^−1^ was able to increase gs regardless of the ECw level. The highest estimated value (0.204 mol H_2_O m^−2^ s^−1^) was obtained in plants irrigated with an ECw of 0.4 dS m^−1^, representing a 23.98% increase compared with plants subjected to the same salinity level but without Se application. The lowest gs (0.137 mol H_2_O m^−2^ s^−1^) was observed in plants exposed to an ECw of 3.1 dS m^−1^ without selenium application (0 mg L^−1^), an effect that was alleviated by applying 11 mg L^−1^ of selenium, which resulted in a 28.83% increase compared with plants not sprayed with Se at the same ECw level.

Regarding transpiration (Figure 2B), the lowest value (1.55 mmol H_2_O m^−2^ s^−1^) was obtained in plants grown under an ECw of 3.1 dS m^−1^, representing a 20.05% reduction compared with those irrigated with 0.4 dS m^−1^. Conversely, selenium application increased E in okra plants, with the highest estimated value (2.25 mmol H_2_O m^−2^ s^−1^) recorded in plants receiving foliar applications of 10 mg L^−1^ Se and irrigated with an ECw of 0.4 dS m^−1^, corresponding to a 16.10% increase compared with the control treatment (no Se application). In addition, selenium exerted a beneficial effect on plants cultivated under the highest salinity level (3.1 dS m^−1^), where the application of 10 mg L^−1^ resulted in an increase of 0.31 mmol H_2_O m^−2^ s^−1^ compared with plants irrigated at the same salinity level but without selenium application.

A similar effect to that observed for stomatal conductance (Figure 2A) and transpiration (Figure 2B) was found for the CO_2_ assimilation rate (Figure 2C), in which selenium application promoted increases in this variable. However, okra plants grown with at estimated concentration of 8.6 mg L^−1^ of Se and irrigated with an ECw of 0.4 dS m^−1^ exhibited the highest A value (15.92 µmol CO_2_ m^−2^ s^−1^), representing an increase of 1.63 µmol CO_2_ m^−2^ s^−1^ compared with plants irrigated under the same salinity level but without Se application. The beneficial effect of applying 8.6 mg L^−1^ of selenium was also observed in okra plants irrigated with an ECw of 3.1 dS m^−1^, increasing the CO_2_ assimilation rate by 1.63 µmol CO_2_ m^−2^ s^−1^ compared with those irrigated under the same conditions but without Se application, which showed the lowest value for this variable (11.38 µmol CO_2_ m^−2^ s^−1^).

**Figure 3:**
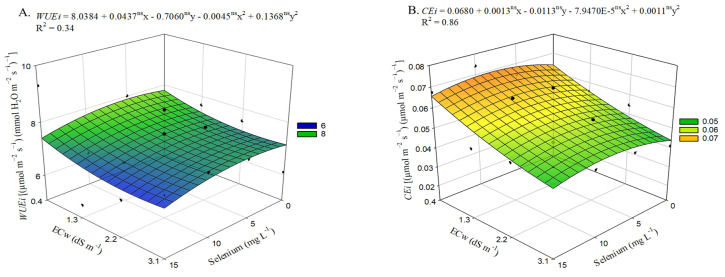
Instantaneous water-use efficiency—*WUEi* (**A**) and instantaneous carboxylation efficiency—*CEi* (**B**) of okra plants as a function of the interaction between irrigation water electrical conductivity and different selenium concentrations, at 65 days after sowing. X and Y—Selenium concentrations and irrigation water electrical conductivity, ns—Not significant by F test. Vertical bars represent the standard error of the mean (*n* = 3).

Selenium application up to estimated concentration of 6.2 mg L^−1^ promoted increases in instantaneous water-use efficiency (Figure 3A), with the highest value of 7.87 served in plants irrigated with an ECw of 0.4 dS m^−1^. This represented a 1.26% increase compared with the control treatment (0 mg L^−1^ Se). In contrast, Se application at 15 mg L^−1^ resulted in the lowest *WUEi* value, 6.81, when plants were irrigated with water at 3.1 dS m^−1^, representing a 4.98% reduction compared with those irrigated under the same conditions but without selenium application.

Selenium application, similar to the effects observed for stomatal conductance (Figure 2A), transpiration (Figure 2B), and CO_2_ assimilation rate, also influenced instantaneous carboxylation efficiency. The highest *CEi* value (Figure 3B), 0.069, was recorded in okra plants subjected at estimated concentration of 8.2 mg L^−1^ of Se and irrigated with an ECw of 0.4 dS m^−1^, representing an 8.36% increase compared with plants irrigated at the same salinity level but without selenium application. The beneficial effect of applying 8.2 mg L^−1^ of Se was also evident in plants irrigated with an ECw of 3.1 dS m^−1^, resulting in a 12.22% increase compared with those grown under the same salinity conditions but without Se application, which exhibited the lowest *CEi* value (0.043).

A significant interaction effect (ECw × Se) was observed for chlorophyll *a* (Chl *a*), total chlorophyll (Chl t), and carotenoid (Car) contents in okra plants. Independently, the levels of electrical conductivity in the irrigation water influenced chlorophyll *b* (Chl *b*), as shown in Table S3.

**Figure 4:**
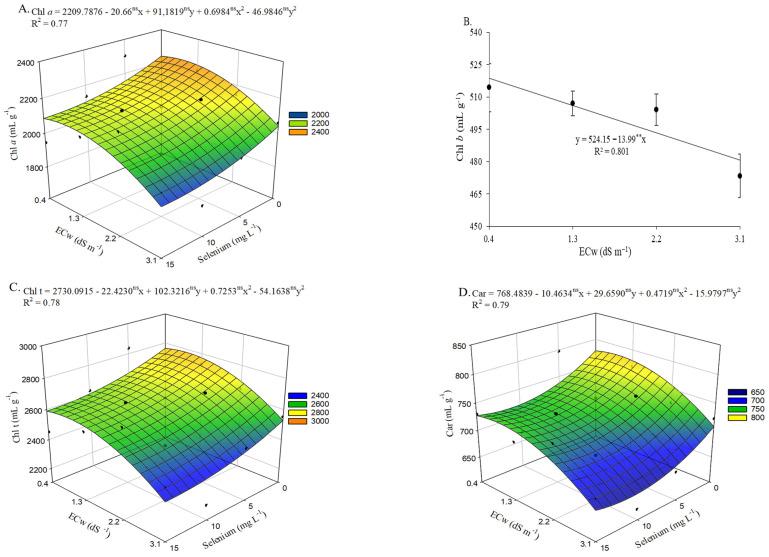
Contents of chlorophyll *a*—Chl *a* (**A**), total chlorophyll—Chl t (**C**), and carotenoids—Car (**D**) of okra plants as a function of the interaction between irrigation water electrical conductivity levels and selenium concentrations, and chlorophyll *b*—Chl *b* (**B**) as a function of irrigation water electrical conductivity levels, at 65 days after sowing. X and Y—Selenium concentrations and irrigation water electrical conductivity, respectively; ** and ns—Significant at *p* ≤ 0.01 and not significant by F test, respectively. Vertical bars represent the standard error of the mean (*n* = 3).

For chlorophyll *a* content (Figure 4A), irrigation with water at 3.1 dS m^−1^ resulted in a reduction of 197.81 mg g^−1^ compared with plants cultivated under an ECw of 0.4 dS m^−1^. The application of selenium, regardless of concentration, reduced the levels of Chl *a*. It was also observed that the lowest value (1888.17 mg g^−1^) occurred in plants irrigated with an ECw of 3.1 dS m^−1^ and subjected to foliar applications of Se at 15 mg L^−1^, representing a 7.48% reduction compared with those that did not receive Se (0 mg L^−1^) and were irrigated at the same salinity level.

Irrigation water salinity caused a linear reduction in chlorophyll *b* levels (Figure 4B) in okra plants, with decreases of 2.67% per each unit increase in ECw. Irrigation with ECw values up to 0.9 dS m^−1^ did not affect total chlorophyll levels (Figure 4C) in okra plants. However, selenium application reduced Chl *b* contents at all concentrations. Plants sprayed with 15 mg L^−1^ of selenium under an ECw of 3.1 dS m^−1^ showed the lowest estimated value (2353.63 mg g^−1^), representing a reduction of 175.15 mg g^−1^ compared with those in the treatment without Se and irrigated at the same salinity level.

As observed for Chl t (Figure 4C), carotenoid levels (Figure 4D) were not affected by irrigation water salinity up to 0.9 dS m^−1^. However, higher ECw levels led to reductions in Car content. Similar to the trends observed for Chl *a* (Figure 4A) and Chl *t (*Figure 4C), the pulverization of selenium intensified the deleterious effects of salinity. When okra plants were sprayed with 15 mg L^−1^ of selenium, reductions were observed, with those irrigated at 3.1 dS m^−1^ showing the lowest estimated value of 656.10 mg g^−1^, reflecting a 7.18% decrease (50.77 mg g^−1^) compared with plants grown under the same salinity level but without Se application (0 mg L^−1^).

There was a significant interaction effect (ECw × Se) on initial chlorophyll a fluorescence (F_0_) and the quantum efficiency of photosystem II (Fv/Fm) (Table S4). Irrigation water salinity levels significantly influenced (*p* ≤ 0.05) maximum fluorescence (Fm) and variable fluorescence (Fv) in okra plants at 65 days after sowing.

**Figure 5:**
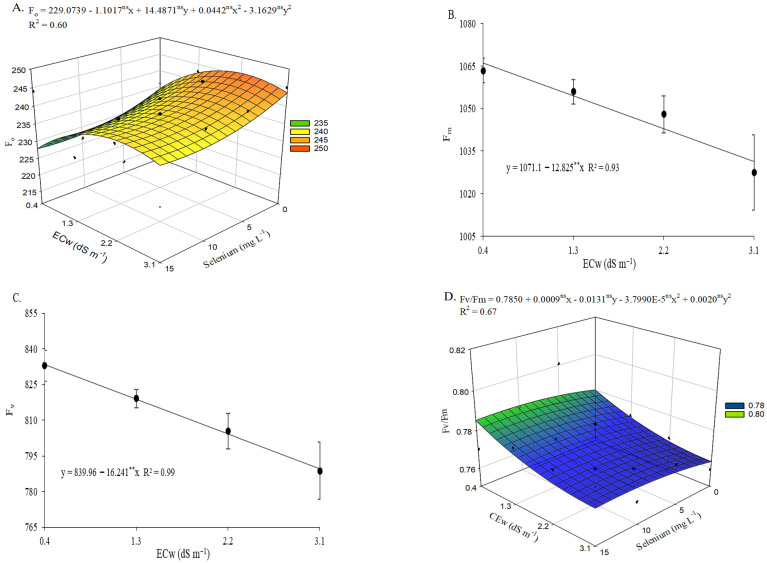
Initial fluorescence—F_o_ (**A**) and quantum efficiency of photosystem II—Fv/Fm (**D**) of okra plants as a function of the interaction between irrigation water electrical conductivity (ECw) levels and selenium concentrations, and maximum fluorescence—Fm (**B**) and variable fluorescence—Fv (**C**) as a function of ECw levels, at 65 days after sowing. X and Y—Selenium concentrations and irrigation water electrical conductivity, respectively; ** and ns—Significant at *p* ≤ 0.01 and not significant by F test, respectively. Vertical bars represent the standard error of the mean (*n* = 3).

Irrigation with brackish water increased initial fluorescence (Figure 5A) in plants cultivated under ECw levels up to 2.3 dS m^−1^, with the highest value (252.66) representing a 4.81% increase compared with plants irrigated with 0.4 dS m^−1^ water and without selenium application. However, the estimated application of 8.2 mg L^−1^ of Se to okra plants irrigated with an ECw of 0.4 dS m^−1^ resulted in the lowest value (228.31), reflecting a 2.59% (6.06) reduction compared with those irrigated under the same salinity level but without Se application (0 mg L^−1^). This concentration mitigated the deleterious effects of salinity in plants grown under the highest water salinity level (3.1 dS m^−1^), reducing F_o_ by 2.49% compared with those irrigated at the same ECw but not subjected to selenium application (0 mg L^−1^).

Maximum fluorescence (Figure 5B) and variable fluorescence (Figure 5C) in okra plants decreased linearly with increasing irrigation water salinity, with reductions of 1.20% and 1.93% per unit increase in ECw, respectively. Regarding the quantum efficiency of photosystem II (Figure 5D), irrigation with water at an ECw of 3.1 dS m^−1^ resulted in a 2.11% reduction compared with plants cultivated under 0.4 dS m^−1^. Conversely, foliar application of selenium at estimated concentrations up to 11.7 mg L^−1^ promoted increases in Fv/Fm, with the maximum value (0.79) recorded in plants irrigated with an ECw of 0.4 dS m^−1^. The beneficial effect of applying 11.7 mg L^−1^ Se was also observed in plants irrigated with an ECw of 3.1 dS m^−1^, resulting in a 1% increase compared with those receiving 0 mg L^−1^ Se, which exhibited the lowest value (0.76).

There was a significant interaction effect (*p* ≤ 0.05) between irrigation water electrical conductivity levels (ECw) and selenium concentrations (Se) on the evaluated growth variables (Table S5).

**Figure 6:**
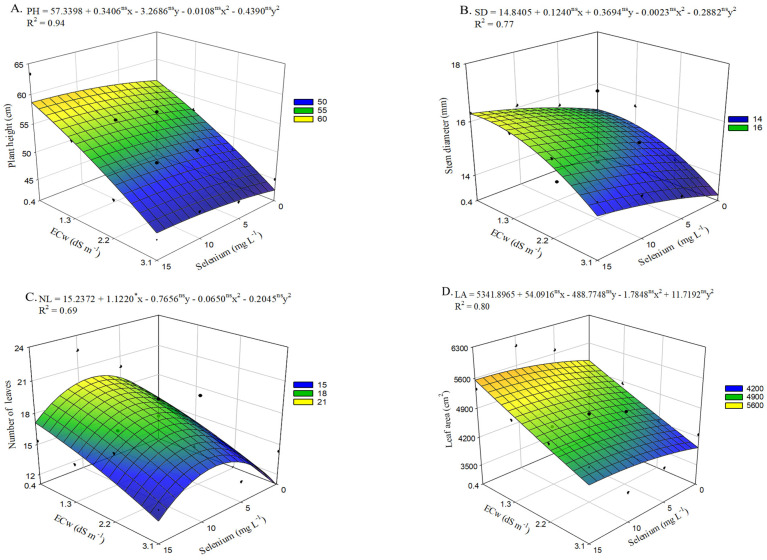
Plant height—PH (**A**), stem diameter—SD (**B**), number of leaves—NL (**C**), and leaf area—LA (**D**) of okra plants as a function of the interaction between irrigation water electrical conductivity levels and selenium concentrations, at 65 days after sowing. X and Y—Selenium concentrations and irrigation water electrical conductivity, respectively; * and ns—Significant at *p* ≤ 0.05 and not significant by F test, respectively. Vertical bars represent the standard error of the mean (*n* = 3).

Regarding plant height (Figure 6A), in the absence of selenium application, plants cultivated under the highest irrigation water salinity level (3.1 dS m^−1^) showed a reduction of 12.97 cm compared with those irrigated with an ECw of 0.4 dS m^−1^. Plants grown under low-salinity irrigation water (0.4 dS m^−1^) and receiving 15 mg L^−1^ of selenium presented the greatest plant height (55.96 cm), reflecting an increase of 2.69 cm relative to the control treatment (ECw of 0.4 dS^−1^ and no Se application). The beneficial effect of Se was also evident in plants irrigated with an ECw of 3.1 dS m^−1^, where the application of 15 mg L^−1^ of Se resulted in a 6.23% increase compared with those irrigated at the same salinity level but without selenium application.

Selenium application at concentrations up to 15 mg L^−1^ increased stem diameter (Figure 6B) in okra plants across all salinity levels. However, the highest estimated value (16.3 mm) was observed in plants cultivated under an ECw of 0.6 dS m^−1^ with the application of 15 mg L^−1^ of Se. When comparing plants irrigated with an ECw of 0.6 dS m^−1^ but without Se application, an increase of 9.09% (1.35 mm) was observed. A similar effect was observed under irrigation with the highest salinity level in the irrigation water, where the concentration of 15 mg L^−1^ resulted in a 10.16% increase in SD compared with plants irrigated at the same ECw level (3.1 dS m^−1^) without application of selenium, which showed the lowest value of 13.21 mm.

Irrigation with the highest salinity level (3.1 dS m^−1^) resulted in the lowest estimated value (10.89 leaves per plant) for the number of leaves (Figure 6C), representing a 26.25% reduction compared with plants cultivated under an ECw of 0.4 dS m^−1^. However, okra plants receiving estimated concentration of 8.6 mg L^−1^ of selenium and irrigated with an ECw of 0.4 dS m^−1^ exhibited the highest NF value (19.75 leaves), corresponding to a 32.57% increase (4.84 leaves) compared with those grown at the same irrigation water conductivity level without selenium application (0.0 mg L^−1^). The beneficial effects of this concentration can also be observed in plants grown under an ECw of 3.1 dS m^−1^, which showed an increase of 44,43% compared with those subjected to the same irrigation water salinity level but sprayed with 0 mg L^−1^ of Se.

Similarly to what was observed for plant height (Figure 6A) and stem diameter (Figure 6B), selenium application at concentrations up to 15 mg L^−1^ promoted an increase in leaf area (Figure 6D). However, the highest estimated value (5558.05 cm^2^) was achieved under low salinity combined with the application of 15.0 mg L^−1^ of Se, representing a 7.96% increase (409.79 cm^2^) compared with plants irrigated under the same conditions but without selenium application. In addition, foliar spraying with 15 mg L^−1^ of Se increased leaf area by 10.40% in plants irrigated with the highest salinity level (3.1 dS m^−1^), compared with those cultivated under the same conditions but without selenium application, which showed the lowest LA value (3939.32 cm^2^).

The interaction between the analyzed factors (ECw × Se) significantly (*p* ≤ 0.05) affected all dry biomass variables of okra plants (Table S6).

**Figure 7:**
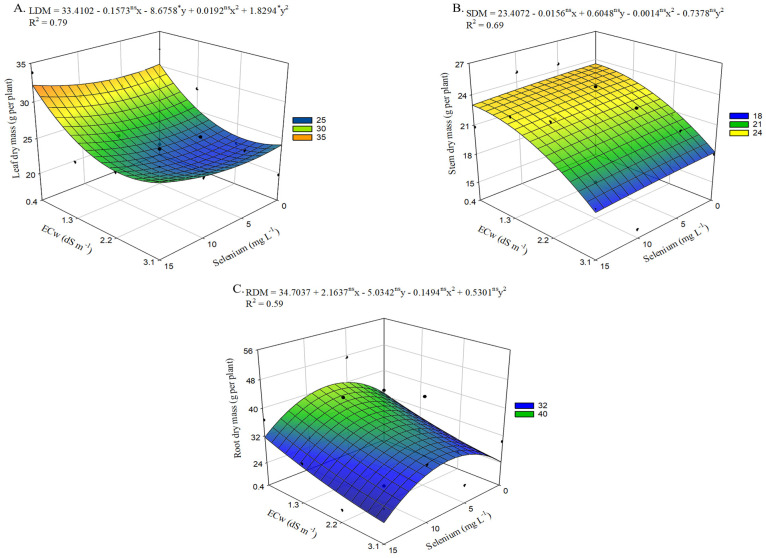
Leaf dry mass—LDM (**A**), stem dry mass—SDM (**B**), and root dry mass—RDM (**C**) of okra plants as a function of the interaction between irrigation water electrical conductivity levels and selenium concentrations, at 94 days after sowing. X and Y—Selenium concentrations and irrigation water electrical conductivity, respectively; * and ns—Significant at *p* ≤ 0.05 and not significant by F test, respectively. Vertical bars represent the standard error of the mean (*n* = 3).

Salinity negatively affected leaf dry mass accumulation (Figure 7A). In plants irrigated with an ECw of 3.1 dS m^−1^, there was a 20.3% reduction in LDM compared with those cultivated under the lowest salinity level (0.4 dS m^−1^). However, selenium application up to 15 mg L^−1^ promoted increases in LDM, with the highest value (32.2 g per plant) observed under an ECw of 0.4 dS m^−1^. The beneficial effect of applying 15 mg L^−1^ of Se was also observed at the highest ECw level, where it increased LDM by 8.14% compared with plants irrigated under the same conditions but without selenium application, which showed the lowest value of 24.09 g per plant.

Stem dry mass (Figure 7B) in okra plants was reduced under ECw levels above 0.4 dS m^−1^, regardless of the selenium concentrations applied. The highest value (23.53 g per plant) was recorded in plants from the control treatment (no Se application). However, selenium application at 15 mg L^−1^ resulted in the lowest value (17.64 g per plant) under irrigation with an ECw of 3.1 dS m^−1^, representing a 3.02% reduction compared with plants under the same water conditions but without Se application.

Selenium application at estimated concentrations up to 7.2 mg L^−1^ increased root dry mass (RDM) (Figure 7C), with the maximum value (40.61 g per plant) observed in okra plants irrigated with an ECw of 0.4 dS m^−1^. This represented a 23.9% increase (7.83 g per plant) compared with plants irrigated at the same salinity level but without Se application. Conversely, concentrations above this threshold resulted in reductions in RDM, with the lowest value (23.03 g per plant) found in okra plants cultivated under an ECw of 3.1 dS m^−1^ and sprayed with 15 mg L^−1^ of Se.

**Figure 8:**
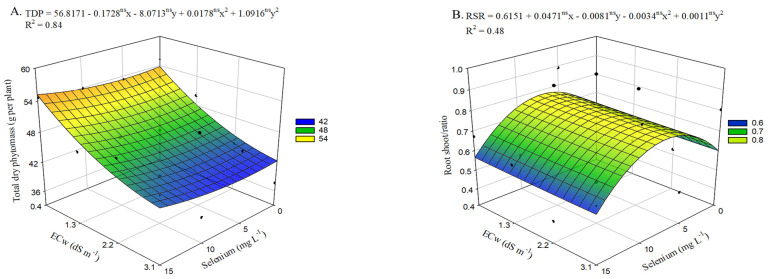
Total dry mass—TDM (**A**) and root-to-shoot ratio—RSR (**B**) of okra plants as a function of the interaction between irrigation water electrical conductivity levels and different selenium concentrations, at 65 days after sowing. X and Y—Selenium concentrations and irrigation water electrical conductivity, ns—Not significant by F test. Vertical bars represent the standard error of the mean (*n* = 3).

Total dry mass (TDM) (Figure 8A) in okra plants not treated with selenium was negatively affected when irrigated with ECw levels above 0.4 dS m^−1^, with a decrease of 21.35% (11.48 g per plant) when comparing the lowest irrigation water electrical conductivity with the highest value (3.1 dS m^−1^). On the other hand, selenium application up to 15 mg L^−1^ increased TDM, with the highest estimated value (55.2 g per plant) observed in plants cultivated under an ECw of 0.4 dS m^−1^, representing a 2.63% increase compared with plants under the same water conditions but without Se application. The highest selenium concentration also promoted increases under high ECw conditions (3.1 dS m^−1^), with an increment of 1.41 g per plant in TDM compared with plants grown under the same salinity conditions but without Se application.

The root-to-shoot ratio (Figure 8B) of plants grown without selenium application decreased as irrigation water salinity increased from 0.4 to 3.1 dS m^−1^, with a decline of 1.90%. However, estimated selenium application at 7.2 mg L^−1^ resulted in the highest RSR value (0.77) in plants irrigated with an ECw of 0.4 dS m^−1^, representing a 26.61% increase compared with plants irrigated under the same electrical conductivity level but without Se application. However, the highest selenium concentration led to reductions in this variable under the highest ECw level, decreasing the RSR of plants sprayed with 15 mg L^−1^ of Se, which showed the lowest value (0.54). This represents a 9.74% reduction compared with those grown under the same salinity level but without Se application (0 mg L^−1^).

A significant interaction effect (*p* ≤ 0.01) was observed between irrigation water electrical conductivity levels and selenium concentrations on the yield components of okra (Table S7).

**Figure 9:**
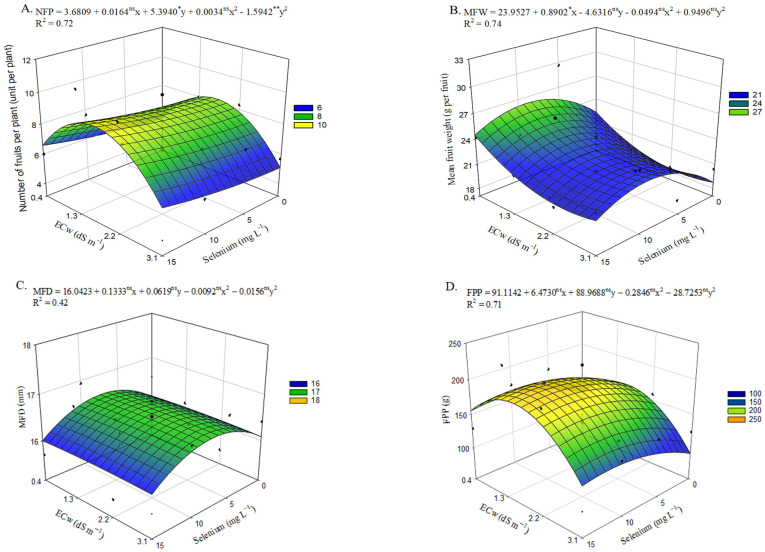
Number of fruits per plant—NFP (**A**), mean fruit weight—MFW (**B**), mean fruit diameter—MFD (**C**), and total fruit production per plant—PFP (**D**) of okra plants as a function of the interaction between irrigation water electrical conductivity levels and selenium concentrations, from 60 to 94 days after sowing. X and Y—Selenium concentrations and irrigation water electrical conductivity, respectively; *, **, and ns—Significant at *p* ≤ 0.05, *p* ≤ 0.01, and not significant by F test, respectively. Vertical bars represent the standard error of the mean (*n* = 3).

Regarding the number of fruits per plant (Figure 9A), the lowest quantity (5.1 fruits per plant) was obtained in plants that did not receive selenium application and were irrigated with an ECw of 3.1 dS m^−1^. However, a beneficial effect on NFP was observed, with the maximum value (9.25 fruits per plant) reached in plants subjected to foliar application of up to 15 mg L^−1^ of Se and irrigated with water at 1.7 dS m^−1^, reflecting a 12.26% increase compared with okra plants irrigated at the same ECw level but without Se application. Beneficial effects of Se application at 15 mg L^−1^ were also observed in plants irrigated with ECw of 3.1 dS m^−1^, with a 19.89% increase in NFP compared with those grown under the same salinity level in the absence of Se.

Mean fruit weight (Figure 9B) was negatively affected by the increase in irrigation water electrical conductivity up to an ECw of 2.4 dS m^−1^, with the lowest value (18.31 g per fruit) representing a 17.72% reduction compared with plants cultivated under the lowest salinity level. On the other hand, an increase in MFW was observed in okra plants subjected to estimated foliar applications of up to 9.0 mg L^−1^ of selenium, which resulted in the highest estimated value (26.26 g per fruit) when irrigated with an ECw of 0.4 dS m^−1^ an increase of 4.04 g per fruit compared with plants grown under the same conditions without Se application. In plants irrigated with an ECw of 2.4 dS m^−1^, the application of 9.0 mg L^−1^ Se contributed to a 21.02% increase in MFW compared with plants cultivated without Se application under the same salinity level.

Similarly to what was observed for MFW (Figure 9B), selenium application at estimated concentrations up to 7.2 mg L^−1^ increased mean fruit diameter (Figure 9C). The maximum value (16.58 mm) was obtained under an irrigation water salinity of 2.0 dS m^−1^, representing a 3.01% increase compared with okra plants irrigated at the same electrical conductivity level without selenium. Conversely, the application of 15 mg L^−1^ of selenium resulted in the lowest value (16.00 mm) when plants were irrigated with an ECw of 3.1 dS m^−1^, reflecting a 0.52% decrease compared with untreated plants grown under the same conditions.

Total fruit production per plant (EPP) (Figure 9D) was affected by the increase in salt concentration in the irrigation water, with the lowest estimated value (90.9 g per plant) obtained in plants irrigated with an ECw of 3.1 dS m^−1^, representing a 25.58% reduction compared with those cultivated under 0.4 dS m^−1^. However, this effect was mitigated by selenium application at estimated concentrations up to 11.4 mg L^−1^. When this concentration was combined with irrigation using an ECw of 2.0 dS m^−1^, the highest estimated value of 196.74 g per plant was observed, corresponding to a 23.01% increase compared with plants that did not receive Se under the same salinity level. The beneficial effect of selenium was also evident in plants cultivated under an ECw of 3.1 dS m^−1^, where the application of 11.4 mg L^−1^ of Se resulted in a 40.50% increase in PFP compared with those that did not receive Se (0 mg L^−1^) and were irrigated under the same conditions.

There was a significant interaction (*p* ≤ 0.01) between the evaluated factors (ECw × Se) for water-use efficiency. Irrigation water salinity levels significantly affected water consumption (Table S8).

**Figure 10:**
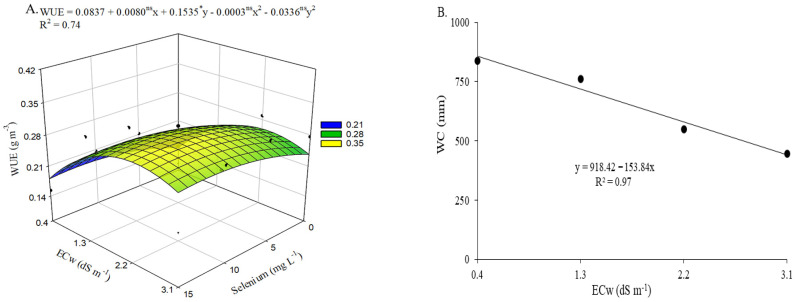
Water-use efficiency—WUE of okra plants as a function of the interaction between irrigation water electrical conductivity levels and selenium concentrations (**A**), and water consumption—WC of okra plants as a function of irrigation water electrical conductivity levels (**B**), from 60 to 94 days after sowing. X and Y—Selenium concentrations and irrigation water electrical conductivity, respectively; * and ns—Significant at *p* ≤ 0.05 and not significant by F test, respectively. Vertical bars represent the standard error of the mean (*n* = 3).

Water use efficiency (Figure 10A) was positively influenced by selenium application at a concentration of 13.3 mg L^−1^. However, the maximum WUE value (0.3123 g m^−3^) was observed in plants irrigated with an ECw of 2.3 dS m^−1^ and subjected to estimated foliar applications of 13.3 mg L^−1^ Se, resulting in a 20.59% increase compared with those grown under the same conditions but without Se application. The estimated concentration also resulted in a 22.54% increase in WUE in okra plants irrigated with the highest ECw, compared with those under the same irrigation conditions but not sprayed with selenium (0 mg L^−1^). The lowest WUE value (0.1397 g m^−3^) was recorded in plants from the control treatment.

Water consumption (Figure 10B) decreased linearly with increasing irrigation water electrical conductivity, with a reduction of 16.75% per each unit increase in ECw. When comparing plants irrigated with water at 3.1 dS m^−1^ to those cultivated under the lowest salinity level (0.4 dS m^−1^), a 48.47% reduction in WC was observed.

**Figure 11:**
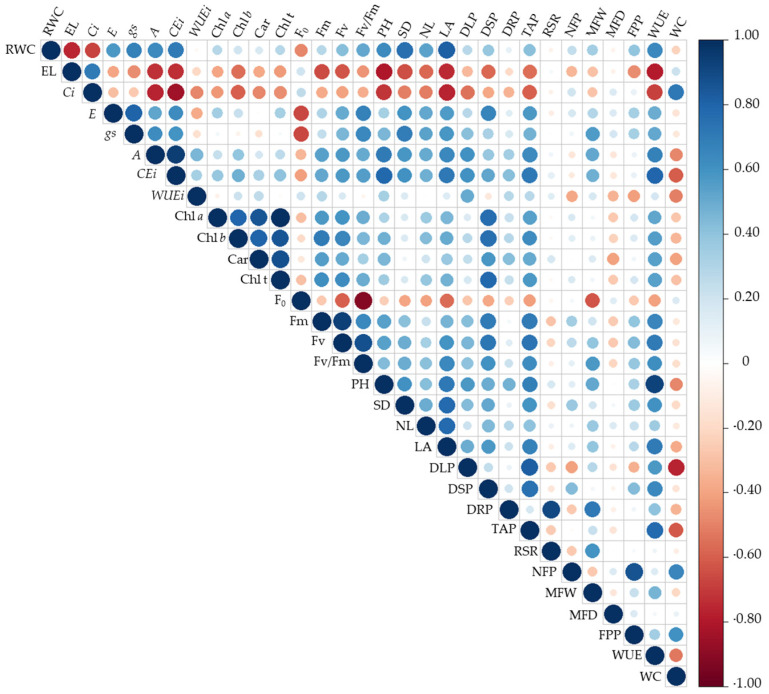
Pearson correlation matrix for the analyzed variables of okra plants as a function of different irrigation water electrical conductivity levels and selenium concentrations. RWC refers to relative water content (%); EL to electrolyte leakage (%); *Ci* to intercellular CO_2_ concentration (μmol CO_2_ m^−2^ s^−1^); *gs* to stomatal conductance (mol H_2_O m^−2^ s^−1^); *A* to CO_2_ assimilation rate (μmol CO_2_ m^−2^ s^−1^); *E* to transpiration (mmol H_2_O m^−2^ s^−1^); *WUEi* to instantaneous water-use efficiency (μmol CO_2_ m^−2^ s^−1^) (mol H_2_O m^−2^ s^−1^)^−1^; Chl *a*, Chl *b*, and Chl *t* to chlorophyll *a*, chlorophyll *b*, and total chlorophyll (mL g^−1^), respectively; F_0_ to initial fluorescence; Fm to maximum fluorescence; Fv to variable fluorescence; Fv/Fm to the quantum efficiency of photosystem II; PH to plant height (cm); SD to stem diameter (mm); NL to number of leaves; LA to leaf area (cm^2^); SDM to stem dry mass (g); TDM to total dry mass (g); NFP to number of fruits per plant; PFP to total fruit production per plant (g); WUE to water-use efficiency (g m^−3^); and WC to water consumption (mm).

Changes in morphophysiological and yield-related variables can be observed in the Pearson correlation matrix (Figure 11). Yield variables showed the lowest correlation coefficients (r < 0.50). However, relative water content, electrolyte leakage, gas exchange, photosynthetic pigments, initial chlorophyll *a* fluorescence, growth components, biomass accumulation, and water consumption exhibited higher correlation indices among themselves.

Electrolyte leakage, internal carbon concentration, and initial chlorophyll *a* fluorescence were negatively correlated (r < −0.50) with all variables, except for water consumption. Strong negative correlations were also observed for RWC, with values of −0.76, −0.70, and −0.50 in relation to EL, Ci, and F_o_, respectively. On the other hand, a positive correlation was found between RWC and gas exchange variables (r > 0.60), as well as growth variables (r > 0.60), except for *WUEi*, which showed a negative correlation. However, no strong correlation was observed among photosynthetic pigments or chlorophyll *a* fluorescence in okra plants.

A positive correlation (r > 0.50) was also observed for *E*, *gs*, *A* and *CEi* with all variables, except for EL, *Ci*, and F_o_, which showed negative correlations < −0.50. Photosynthetic pigment contents were strongly correlated with each other (r > 0.80) and also with maximum and variable fluorescence, which displayed correlations above 0.50. Mean fruit weight (MFW) showed positive correlations above 0.50 with gas exchange variables, except for internal carbon concentration. However, it exhibited a lower correlation with F_0_ (−0.67). Mean fruit weight also showed a positive correlation (0.60) with Fv/Fm, an effect possibly associated with greater photosynthetic efficiency.

WUE exhibited positive correlations (r > 0.50) with all variables except electrolyte leakage, internal CO_2_ concentration, and initial chlorophyll a fluorescence, which showed correlations of −0.79, −0.70, and −0.41, respectively. Water consumption showed a strong positive correlation only with Ci and NFP (r > 0.60), while correlating negatively with the remaining variables.

**Figure 12:**
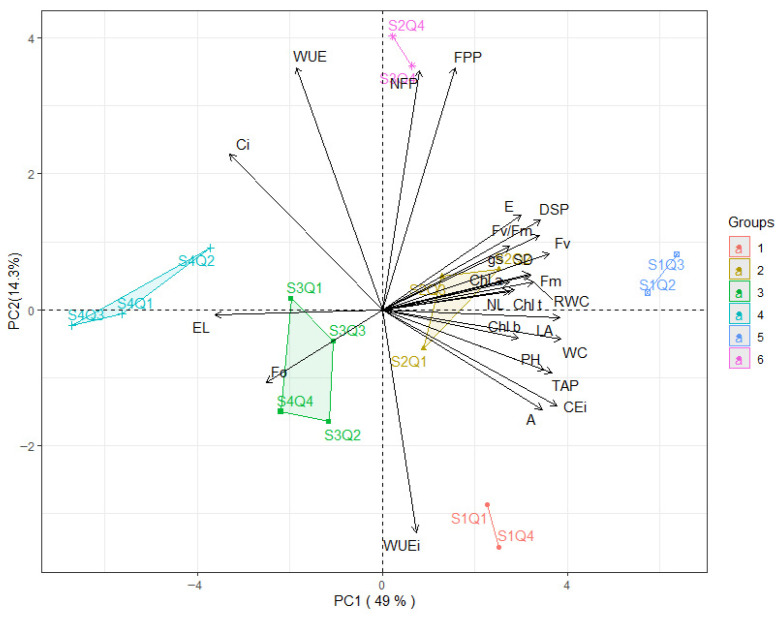
Two-dimensional projection of principal component scores for irrigation water electrical conductivity levels (ECw), selenium concentrations (Se), and the analyzed variables of okra plants in the two principal components (PC1 and PC2). RWC refers to relative water content (%); EL to electrolyte leakage (%); Ci to intercellular CO_2_ concentration (μmol CO_2_ m^−2^ s^−1^); *gs* to stomatal conductance (mol H_2_O m^−2^ s^−1^); *A* to CO_2_ assimilation rate (μmol CO_2_ m^−2^ s^−1^); *E* to transpiration (mmol H_2_O m^−2^ s^−1^); *WUEi* to instantaneous water-use efficiency (μmol CO_2_ m^−2^ s^−1^) (mol H_2_O m^−2^ s^−1^)^−1^; Chl *a*, Chl *b*, and Chl *t* to chlorophyll *a*, chlorophyll *b*, and total chlorophyll (mL g^−1^), respectively; F_0_ to initial fluorescence; Fm to maximum fluorescence; Fv to variable fluorescence; Fv/Fm to the quantum efficiency of photosystem II; PH to plant height (cm); SD to stem diameter (mm); NL to number of leaves; LA to leaf area (cm^2^); SDM to stem dry mass (g); TDM to total dry mass (g); NFP to number of fruits per plant; PFP to total fruit production per plant (g); WUE to water-use efficiency (g m^−3^); and WC to water consumption (mm). S1Q1 (0.4 dS m^−1^ and 0 g L^−1^); S1Q2 (0.4 dS m^−1^ and 5 g L^−1^); S1Q3 (0.4 dS m^−1^ and 10 g L^−1^); S1Q4 (0.4 dS m^−1^ and 15 g L^−1^); S2Q1 (1.3 dS m^−1^ and 0 g L^−1^); S2Q2 (1.3 dS m^−1^ and 5 g L^−1^); S2Q3 (1.3 dS m^−1^ and 10 g L^−1^); S2Q4 (1.3 dS m^−1^ and 15 g L^−1^); S3Q1 (2.2 dS m^−1^ and 0 g L^−1^); S3Q2 (2.2 dS m^−1^ and 5 g L^−1^); S3Q3 (2.2 dS m^−1^ and 10 g L^−1^); S3Q4 (2.2 dS m^−1^ and 15 g L^−1^); S4Q1 (3.1 dS m^−1^ and 0 g L^−1^); S4Q2 (3.1 dS m^−1^ and 5 g L^−1^); S4Q3 (3.1 dS m^−1^ and 10 g L^−1^) and S4Q4 (3.1 dS m^−1^ and 15 g L^−1^).

The interaction between irrigation water electrical conductivity levels (ECw) and selenium concentrations (Se) can be observed in the principal component analysis (Figure 12). The PCA explained 63.30% of the original variation, corresponding to the two components (PC1 and PC2), which individually accounted for 49.0% and 14.30% of the variance, respectively.

For the first component, positive correlations were observed for RWC (0.72), *E* (0.65), *gs* (0.61), *A* (0.79), *CEi* (0.87), Chl *a* (0.63), Chl *b* (0.68), Chl t (0.66), Fm (0.73), Fv (0.83), Fv/Fm (0.77), PH (0.82), SD (0.69), NL (0.62), LA (0.86), SDM (0.77), TDM (0.85), and WC (0.90), which were favored by groups 2 and 5. In contrast, negative correlations were found for EL (−0.80), *Ci* (−0.76), and F_o_ (−0.60), a pattern associated with groups 3 and 4.

For the components of axis 2, positive correlations were observed for NPF (0.84), PFP (0.80), and WUE (0.76), which were associated with group 6. A negative correlation was observed only for *WUEi* (−0.73), a behavior influenced by grouping 1.

## 3. Discussion

The semiarid region of Northeastern Brazil is characterized by an uneven distribution of rainfall in both time and space, combined with high evapotranspiration rates, which favors water scarcity and the accumulation of salts in the soil solution and water sources [18]. Over the past decade, the expansion of salinized areas and the presence of high concentrations of dissolved salts in water sources have posed a significant threat to agriculture due to their effects on plant growth, productivity, and product quality [19]. Salt stress is considered one of the abiotic stresses affecting several crops, representing a major challenge for crop production in semiarid regions such as Northeastern Brazil [20]. However, foliar application of certain substances may be a promising strategy to enhance plant tolerance to salinity, and one such substance is selenium (Se) [17].

In the present study, reductions were observed in the variables relative water content of the leaf blade, leaf gas exchange, photosynthetic pigments, chlorophyll *a* fluorescence, growth, yield, and water use efficiency of okra plants irrigated with brackish water. The reduction in relative water content (Figure 1A) is possibly associated with the decrease in soil solution osmotic potential due to the accumulation of soluble salts in the rhizosphere [21], thereby affecting water and nutrient uptake and leading to reduced cellular turgor.

With reduced turgor, cells become flaccid, which may favor the release of ions when subjected to increased turgor pressure. The lower cellular turgor caused by salt stress likely resulted in membrane damage, leading to an increase in EL (Figure 1B), since values above 40% are considered indicative of severe injury to this structure [22]. This effect is possibly associated with the induction of oxidative stress resulting from the excessive production of reactive oxygen species [23]. These conditions promote membrane lipid peroxidation, thereby compromising membrane integrity. Reinforcing the antagonistic relationship between cellular turgor and EL in plants under salt stress, a strong negative correlation between these variables was observed (Figure 11).

However, selenium application at a concentration of 15 mg L^−1^ mitigated these effects in plants cultivated under an ECw of 3.1 dS m^−1^. Previous studies have also reported the beneficial effects of selenium in common bean (*Phaseolus vulgaris* L.) plants grown under salt stress (0 and 50 mM). Farag et al. [12] observed that Se applied at concentrations of 5 or 10 µM increased RWC by 8.5% and 3.2%, respectively, compared with plants that did not receive Se under saline conditions at the corresponding concentrations.

The increase in RWC in okra plants may be associated with the role of selenium in promoting the accumulation of protective osmolytes in plant cells and enhancing root performance in water absorption, thereby contributing to better cellular maintenance [7]. This effect may also be related to a possible regulation of the K^+^/Na^+^ balance in plant cells, leading to improved cellular osmotic conditions and better conservation of intracellular water [24]. Another noteworthy point is that selenium application reduced electrolyte leakage in the leaf blade (Figure 1B) of okra plants. This phenomenon may be attributed to the essential physiological roles of selenium, whose primary function is as an antioxidant, crucial for protecting plant tissues [25], thereby reducing electrolyte leakage.

Despite the coordinated role of selenium in antioxidant activity and osmotic adjustment which helps maintain membrane integrity [26] high Se concentrations caused cellular injury (Figure 2B) when combined with higher ECw levels. Elevated Se levels can disrupt protein structure and function by displacing cysteine and methionine, resulting in toxic effects [27]. In addition, the excessive accumulation of reactive oxygen species (ROS) induced by selenium may intensify its toxicity, leading to cellular damage [28].

With increasing salinity in the irrigation water, a reduction in gas exchange variables was observed. These results may be associated with the decline in RWC (Figure 1A) caused by the higher salt concentration in the irrigation water, which favors partial stomatal closure, an effect supported by the strong positive correlation shown in the Pearson matrix (Figure 11) among these variables. Commonly, under such conditions, these res-ponses are associated with a plant defense mechanism against stress, in which stomatal closure occurs to prevent excessive water loss through transpiration to the atmosphere [29], due to increased levels of abscisic acid (ABA), which contributes to the adjustment and stabilization of the plant’s water potential [30].

With reduced *gs* (Figure 2A), the diffusion of CO_2_ from the atmosphere into the substomatal chambers decreases, directly affecting the Calvin-Benson cycle, which lowers instantaneous carboxylation efficiency (Figure 3A) as irrigation water salinity increases. In the absence of sufficient CO_2_, oxygen binds to the enzyme ribulose−1,5-bisphosphate carboxylase/oxygenase (RuBisCO), triggering a possible photorespiratory process that directly affects the production of photoassimilates and secondary carbon metabolism [31].

However, these effects were alleviated by selenium application, as reflected in *gs* (Figure 2A), *E* (Figure 2B), *A* (Figure 2C), and *CEi* (Figure 3B) of plants irrigated with brackish water. These increases may be associated with the protective effect provided by exogenous selenium applied at low concentrations, primarily due to the reduction of reactive oxygen species [32]. Selenium, in its selenate form, can act to stabilize the electron transport process during photosynthesis, preventing the accumulation of reactive oxygen species and thus offering greater protection, functionality, and integrity to chloroplasts [33], preserving the structure and function of the photosynthetic apparatus. In addition, Se may act in various physiological processes that maintain cellular structure and function, contributing to plant growth and development [34].

Irrigation water salinity negatively affected the levels of photosynthetic pigments, including chlorophyll *a* (Figure 4A), chlorophyll *b* (Figure 4B), total chlorophyll (Figure 4C), and carotenoids (Figure 4D). These results suggest that the excess salts in the irrigation water compromise the integrity of photosystem II, causing a reduction in the conversion of light energy into chemical energy by the antenna complex.

The decline in the synthesis of photosynthetic pigments under saline conditions may be linked to chloroplast degradation and/or increased activity of the chlorophyllase enzyme, promoting chlorophyll photo-oxidation and triggering a reduction in antenna complex efficiency [35]. This process increases the energy demand for electron flow to plastoquinone and subsequently to the electron transport chain, resulting in lower ATP and NADPH^+^ production for the Calvin cycle [36].

Selenium application may contribute to the induction of tolerance by protecting photosynthetic pigments from oxidative damage, thereby improving photosynthetic efficiency and acting directly in preserving chlorophyll content and mitigating oxidative damage under stress conditions [37]. In addition, it helps preserve carotenoids, enhancing photosynthetic efficiency and preventing photodamage [38].

However, in the present study, exogenous selenium application intensified the de-leterious effects of irrigation water salinity. This response may be associated with the ne-gative impact of excessively high selenium doses, which can cause injuries to physiological and biochemical variables, indicating the potential toxicity of excessive Se application. Such toxicity can disrupt the balance of other essential nutrients, leading to growth inhibition and physiological damage to plants [39].

Okra plant growth was affected by the increase in irrigation water salinity, particularly when plants were irrigated with 3.1 dS m^−1^. Salt stress inhibits plant growth due to increased metabolic energy demands, resulting from reduced carbon assimilation, decreased photosynthetic efficiency per unit leaf area, disruption of carbohydrate and protein metabolism, and impaired water uptake [40]. These factors also compromise cellular differentiation processes, limiting plant growth in terms of height, stem diameter, and leaf expansion [41].

However, it was observed that selenium concentrations between 8.6 and 15.0 mg L^−1^ mitigated the effects of irrigation with brackish water up to an ECw of 3.1 dS m^−1^. Similar results were reported by Wu et al. [13] when evaluating selenium application (25 μM) in tomato plants (*Solanum lycopersicum*) irrigated with 0 and 150 mM NaCl. They found that Se application increased plant height and stem diameter by 17.7% and 23.30%, respectively, under salt stress compared with the control treatment. Likewise, Shalaby et al. [14], when evaluating selenium application (25 mg L^−1^) in cucumber (*Cucumis sativus*) grown in soils with high dissolved salt content (ECw = 4.49 dS m^−1^) during different seasons (warm and cold), reported that Se application increased plant height to 286.00 cm and 355.00 cm and leaf area to 79.65 and 96.40 dm^2^ in both periods, respectively, compared with the control.

The growth promoting effects observed in plants subjected to selenium application may be associated with increased starch accumulation in chloroplasts, providing protection to cellular components and stimulating cell division and elongation, which results in greater plant height and larger leaf area as observed in this study [42]. In addition, Se acts in several physiological processes that maintain cellular structure and function, contributing to plant growth and development [43].

Okra yield was significantly reduced by the increase in salt concentration in the irrigation water. Similar results were found by Veloso et al. [44], who reported a 41.78% decrease (4 fruits per plant) under an ECw of 3.2 dS m^−1^ compared with plants irrigated with an ECw of 0.8 dS m^−1^. Salt stress can inhibit leaf expansion by reducing turgor, leading to declines in photosynthesis and growth, ultimately affecting crop yield [45]. According to Ferreira et al. [46], the decrease in these variables may be associated with limitations in gas exchange, resulting in reduced translocation of photoassimilates and, consequently, restricted fruit development.

On the other hand, selenium application at concentrations ranging from 7.2 to 15 mg L^−1^ increased the number of fruits (Figure 9A), mean fruit weight (Figure 9B), mean fruit diameter (Figure 9C), and total fruit production per plant (Figure 9D) across all salinity levels. Similar results were reported by Pourebrahimi et al. [47], who evaluated the effect of selenium application at 1.0 mg L^−1^ in strawberry cultivation under saline irrigation (0 to 40 mM NaCl) in a hydroponic system. They found that Se application increased fruit length and fruit diameter by 43.00% and 52.00%, respectively, when plants were irrigated under saline conditions compared with the control treatment. 

With the greater cellular turgidity observed (Figure 1A), the processes of cell expansion and division become more active, which favors increased growth and a higher number of leaves (Figure 6C) and leaf area (Figure 6D). This increase in photosynthetically active area is consistent with the positive correlation between relative water content and growth variables shown in Figure 11. The expansion of leaf area, associated with greater cellular turgor, promoted stomatal opening (Figure 2A), intensifying transpiration (Figure 2B) and CO_2_ diffusion into the substomatal chambers, which may have enhanced the photosynthetic rate (Figure 2C). These results indicate that these variables are directly proportional and contribute to greater plant growth and development, resulting in increased availability of photoassimilates allocated to the fruits.

An important aspect is that water-use efficiency was positively influenced by selenium application. This effect may be associated with greater cellular turgor promoted by more efficient osmotic adjustment and increased transpiration, both of which favored water uptake by the plants [7,12,29]. Thus, even under saline conditions, the plants were able to maintain growth and yield.

**Figure 13:**
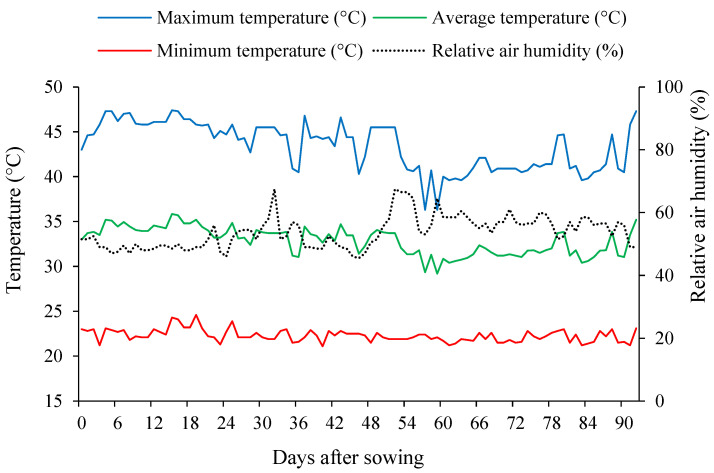
Maximum, mean, and minimum air temperatures and mean relative air humidity inside the greenhouse during the experimental period (23 March to 25 June 2025).

An important point to highlight is that the high temperatures typical of the semiarid region of Northeastern Brazil can negatively affect okra production. The crop prefers temperatures between 21.1 °C and 29.4 °C, with tolerance to maximum temperatures close to 35 °C and minimum temperatures of 18.3 °C [48]. However, as shown in Figure 13, the recorded values exceeded the upper limit considered adequate for okra cultivation. According to Ghani et al. [49], heat stress in semiarid regions intensifies photochemical activity and physiological drought in plants, causing disturbances in the production of reactive oxygen species, which affect oxidative homeostasis and, consequently, plant development.

When heat stress occurs during the reproductive stage, it may compromise pollen viability, reducing fruit set [50]. Formagio et al. [51] also report that thermal fluctuations during flowering can cause malformations in the pollen tube, as well as alterations in style curvature and nectary length traits directly related to fruit formation and quality.

However, these effects can be mitigated by selenium application, as its use can enhance the antioxidant capacity of stressed plants. Exogenous selenium application may influence plant metabolism at most growth stages, leading to statistically significant increases in biomass and higher fruit yields [52]. Se can act directly by strengthening antioxidant defense systems, improving photosynthetic efficiency, and enhancing secondary carbon metabolism, thereby increasing plant resilience to abiotic stresses [10].

However, it is important to emphasize that excessive selenium application can lead to phytotoxicity. This effect is attributed to the ability of the element to accumulate in plant tissues through sulfate transporters and subsequently be incorporated into selenocysteine (Se-Cys) and selenomethionine (Se-Met) via the sulfur assimilation pathway [53]. Selenium-induced phytotoxicity results primarily from the nonspecific substitution of Cys and/or Met with these analogs in polypeptide chains, causing structural and functional disruption of proteins [54].

Selenium supplementation at concentrations of 2 and 6 mg L^−1^ has been associated with increased photosynthetic rate, acting as an antioxidant agent. However, at higher concentrations, such as 10 mg L^−1^, the authors highlighted that Se may inhibit the photosynthetic process, promoting damage to the photosynthetic apparatus [55]. According to Smolén et al. [56], this toxic effect occurs because higher doses of selenium distort protein structure and functions.

Thus, establishing an appropriate supplementation threshold is essential, since elevated concentrations may cause severe injuries to plants.

## 4. Materials and Methods

The experiment was conducted from March 23 to June 25 in a greenhouse belonging to the Academic Unit of Agricultural Engineering (UAEA) at the Federal University of Campina Grande (UFCG), located in Campina Grande city, PB, Brazil, at coordinates 7° 15′ 18″ S and 35° 52′ 28″ W, with an average altitude of 550 m. The structure used was an arch-type greenhouse, 30 m long, 21 m wide, with a 3.0 m sidewall height, and covered with low-density polyethylene (150 µm). Air temperature (maximum, mean, and minimum) and relative air humidity inside the greenhouse during the experimental period are presented in Figure 13, with mean values of 32.84 °C and 53.71% for temperature and relative air humidity, respectively.

The treatments consisted of the combination of four irrigation water salinity levels, expressed as the water electrical conductivity—ECw (0.4, 1.3, 2.2, and 3.1 dS m^−1^), and four selenium concentrations—Se (0, 5, 10, and 15 mg L^−1^), arranged in a randomized block design in a 4 × 4 factorial scheme, with three replicates and one plant per plot. The irrigation water electrical conductivity levels were established based on the study by Soares et al. [57], conducted with okra plants, while the selenium concentrations were defined according to the methodology proposed by Amerian et al. [17] in an experiment with cucumber irrigated with brackish water.

The plants were grown in 20 L (with a diameter of 31 cm and a height of 35 cm) pots adapted as drainage lysimeters. Each pot was perforated at the base and connected to a transparent drain, over which a geotextile fabric and a 0.3 kg layer of crushed stone (N^o^ 0) were placed to prevent clogging by the soil. The drained water was collected in 2 L polyethylene bottles positioned beneath each drain, allowing the determination of water consumption by okra plants throughout the cycle.

The soil used in the experiment was classified as a *Neossolo Regolítico* (Entisol), with a sandy loam texture, collected in the municipality of Riachão do Bacamarte, PB, Brazil, located at 7° 10′ 08″ S and 35° 51′ 20″ W, at a depth of 0–30 cm. The chemical and physical hydraulic properties of the soil were determined according to the methodology described by Teixeira et al. [58].

The soil analysis revealed a pH of 5.40. Organic matter (OM) content was 17.42 g dm^−3^, while available phosphorus (P) was 2.92 mg dm^−3^. Exchangeable cation contents were as follows: potassium (K), 0.28 cmolc kg^−1^; sodium (Na), 0.04 cmolc kg^−1^; calcium (Ca^2+^), 1.87 cmolc kg^−1^; and magnesium (Mg^2+^), 1.70 cmolc kg^−1^. Acidity-related elements included aluminum (Al^3+^) at 0.20 cmolc kg^−1^ and hydrogen plus aluminum (H^+^ + Al^3+^) at 2.88 cmolc kg^−1^. With respect to physical properties, particle-size analysis showed the following composition: 675.2 g kg^−1^ sand, 221.8 g kg^−1^ silt, and 103 g kg^−1^ clay. Soil bulk density was 1.51 g cm^−3^. Soil moisture content was 5.34 dag kg^−1^, and the maximum compaction pressure reached was 33.42 kPa.

Okra seeds of the cultivar Santa Cruz 47 were used, characterized by being a traditional open pollinated variety. This cultivar produces cylindrical, pointed fruits with a bright green color, no visible fibers in the pulp, lengths ranging from 10 to 18 cm, and a commercial diameter of 1.5 cm. Four seeds were sown per pot, evenly spaced, at a standard depth of approximately 3 cm, and thinning was performed five days after sowing.

Nitrogen fertilization (urea-45% N), phosphorus (monoammonium phosphate—60% P_2_O_5_ and 12% N), and potassium (potassium chloride-51.5% K_2_O and 17% S) fertilization began 12 days after sowing (DAS), with the application of 100 mg N, 300 mg P_2_O_5_, and 150 mg K_2_O, respectively. Fertilization was carried out biweekly via fertigation, fo-llowing the recommendations of Novais et al. [59] for pot experiments. Micronutrients were supplied through foliar sprays applied every 15 days, using Dripsol Micro^®^ solution at a concentration of 1.0 g L^−1^ containing (1.2% Mg, 0.85% B, 3.4% Fe, 4.2% Zn, 3.2% Mn, 0.5% Cu, 0.06% Mo) and applied with a backpack sprayer.

Irrigation with brackish water began at 22 DAS. The saline solutions were prepared by dissolving NaCl, CaCl_2_·2H_2_O, and MgCl_2_·6H_2_O in the local supply water (0.4 dS m^−1^), following a 7:2:1 ratio, which is predominant in major irrigation water sources in Northeastern Brazil [60]. The relationship between irrigation water electrical conductivity (ECw) and salt concentration was established according to Richards [61].

After preparation, the saline solutions were calibrated by measuring electrical conductivity using an electronic conductivity meter (TECNOPON^®^). Until 21 days after sowing (DAS), irrigation was performed exclusively with local supply water (0.4 dS m^−1^). From that point onward, the different irrigation water electrical conductivity levels (0.4 and 3.0 dS m^−1^) were established.

Irrigation was performed daily, with the volume of water applied to each pot calculated based on the water balance of the root zone, determined using the drainage lysimetry method, according to the procedure described by Bernardo et al. [62].

The volumes of water used for irrigation during the experiment were recorded to determine plant water consumption, as shown in Table 1.

Sodium Selenate^®^ Anhydrous P.A. (Na_2_SeO_4_) with 99.6% purity was used, and at the time of application, the required concentrations were weighed and diluted in distilled water. Foliar applications began at 21 DAS and were carried out in the afternoon (17:00 h), with repetitions every 15 days, totaling three applications throughout the crop cycle. The adaxial and abaxial leaf surfaces were sprayed using a handheld sprayer. For the control treatments (0 mg L^−1^), spraying was performed using only distilled water. When applying selenium, the volume used was recorded according to each treatment (Table 2) where it varied according to the growth of the okra plants.

At 65 days after sowing, to evaluate the effect of selenium concentrations, morphophysiological assessments were performed on okra plants, including the variables relative water content (RWC), electrolyte leakage (EL), gas exchange, photosynthetic pigments, chlorophyll *a* fluorescence, and growth. Biomass was evaluated at 94 days after sowing, while fruit yield was assessed from 60 to 94 DAS, and water-use efficiency was evaluated throughout the entire crop cycle.

To determine relative water content (RWC), leaves were collected from okra plants, and five leaf discs with an area of 113 mm^2^ were removed. Immediately after collection, the discs were weighed to avoid moisture loss, obtaining the fresh mass (FM). The samples were then immersed in 50 mL of distilled water, placed in beakers, and kept for 24 h. After this period, excess surface water was removed using paper towels, and the turgid mass (TM) was determined. Subsequently, the samples were placed in an oven at appro-ximately 65 ± 3 °C until reaching constant weight, to obtain the dry mass (DM). RWC was determined according to the methodology of Weatherley [63].

The electrolyte leakage, used as an indicator of cell membrane integrity under salt stress conditions, was evaluated by assessing membrane disruption. Five leaf discs, each one with an area of 113 mm^2^, were collected and previously washed with distilled water to remove electrolytes adhered to the surface. The discs were then placed in beakers containing 50 mL of distilled water, which were hermetically sealed with aluminum foil. The containers remained in a chamber at 25 °C for 24 h, after which the initial electrical conductivity (Ci) was measured. The beakers were subsequently transferred to a forced-air oven at 80 °C for 150 min. After this period, the final electrical conductivity (Cf) was measured using a conductivity meter. Electrolyte leakage was then calculated accor-ding to the methodology described by Scotti-Campos et al. [64].

Gas exchange measurements were performed on previously identified leaves selected for their greater representativeness of photosynthetic activity through a reading per plant. Measurements were taken in the morning, between 6:00 and 9:00 a.m. The following variables were determined: stomatal conductance (*gs*, mol H_2_O m^−2^ s^−1^), CO_2_ assimilation rate (*A*, µmol CO_2_ m^−2^ s^−1^), transpiration (*E*, mmol H_2_O m^−2^ s^−1^), and intercellular CO_2_ concentration (*Ci*, µmol CO_2_ m^−2^ s^−1^). Based on these data, instantaneous water-use efficiency (*WUEi* = *A*/*gs*) [(µmol m^−2^ s^−1^) (mmol H_2_O m^−2^ s^−1^)^−1^] and instantaneous carboxylation efficiency (*CEi* = *A*/*Ci*) [(µmol m^−2^ s^−1^) (µmol m^−2^ s^−1^)^−1^] were calculated.

Gas exchange measurements were conducted under an average irradiance of 882.00 µmol photons m^−2^ s^−1^ and considering the approximate photoperiod of okra of 12 h [65]. These evaluations were performed using a portable gas exchange system equipped with an infrared gas analyzer (IRGA), model LCpro-SD (ADC Bioscientific, Hoddesdon, UK).

To determine the levels of photosynthetic pigments, leaf samples were collected from the middle third of the plants and subsequently taken to the Plant Physiology Laboratory of the Federal University of Campina Grande, Campina Grande campus. In these samples, chlorophyll total, chlorophyll *a*, chlorophyll *b*, and carotenoids (Car) were quantified, through a reading per plant. Extraction was performed using dimethyl sulfoxide (DMSO), following the methodology described by Barnes et al. [66] and adapted by Cruz et al. [67].

A leaf disc of known area (113 mm^2^) was used, and each disc was placed in containers with 5 mL of DMSO. The containers were stored in a Styrofoam box to prevent pigment degradation and kept in the dark for 48 h prior to reading. Absorbance measurements were performed using a spectrophotometer at wavelengths of 665, 649, and 480 nm, corresponding respectively to chlorophyll *a*, chlorophyll *b*, and carotenoids. The values obtained for chlorophyll *a*, *b*, total chlorophyll, and carotenoids (Car) in the plants were expressed in mL g^−1^ of fresh matter.

Chlorophyll *a* fluorescence analyses were performed on the same leaves used for gas exchange measurements through a reading per plant. The following variables were determined: initial fluorescence (F_0_), maximum fluorescence (Fm), variable fluorescence [Fv = (Fm − F_0_)], and the maximum quantum efficiency of photosystem II [(Fv/Fm) = (Fm − F_o_)/Fm], using a PEA—Hansatech fluorometer [68]. These measurements were conducted by placing leaf clips on the leaves and, after a 30 min dark adaptation period, recording the values for each variable.

For the growth variables, plant height was measured using a measuring tape, from the base of the stem to the tip of the apical bud; stem diameter was determined using a digital caliper; and leaf area was measured following the methodology proposed by Fideles Filho et al. [69].

The biomass variables analyzed were leaf dry mass (LDM), stem dry mass (SDM), root dry mass (RDM), and total dry mass (TDM), obtained as the sum of LDM and SDM; as well as the root to shoot ratio. For assessing fruit yield of okra plants, harvesting was carried out every two days throughout the reproductive and fruit maturation stages. The following variables were measured: number of fruits per plant (NFP); equatorial fruit diameter (EFD), measured using a digital caliper; mean fruit weight (MFW); total fruit yield per plant (FPP), obtained as the sum of the weight of all fruits per plant; and fruit length (MFD), measured using a graduated ruler.

Water use efficiency (WUE, g m^−3^) was determined as the ratio between the total fruit yield per plant (g plant^−1^) and the water consumption per plant (m^3^), according to the methodology proposed by Guan et al. [70].

The data were subjected to tests for normality (Shapiro–Wilk) and homogeneity of variances (Levene). When significance was detected (*p* ≤ 0.05), an F test was applied for the ECw and selenium concentration factors. When a significant effect was identified, li-near and/or quadratic regression models were fitted using the RStudio statistical software (version 4.1.0). In cases where a significant interaction between factors occurred, response surface plots were generated using SigmaPlot (version 14.5).

Subsequently, when the data met the normality assumption, Pearson’s correlation analysis (*p* ≤ 0.05) was performed for the evaluated variables. A principal component ana-lysis was also conducted, retaining only variables with correlation coefficients above 0.60 [71]. All statistical analyses were performed using RStudio (version 4.1.0), with the support of the AgroR, ggcorrplot, and mclust packages.

## 5. Conclusions

Irrigation with water of electrical conductivity above 0.4 dS m^−1^ reduces the relative water content, gas exchange, growth, chlorophyll *a* fluorescence, fruit yield, and water use efficiency, while increasing electrolyte leakage in the leaf blade. Water salinity higher than 1.0 dS m^−1^ inhibits the synthesis of photosynthetic pigments. The application of selenium did not mitigate the deleterious effects of salinity on the contents of Chl *a*, chlorophyll total, and carotenoids in okra plants irrigated with saline water. However, when applied at concentrations between 8.6 and 15 mg L^−1^, selenium promotes increases in gas exchange, chlorophyll *a* fluorescence, growth, and fruit yield of okra plants irrigated with water of up to 3.1 dS m^−1^. Based on the results obtained, the initial hypothesis that foliar application of selenium mitigates the deleterious effects of salinity on the physiology, growth, and yield of okra plants was confirmed. However, the effects of selenium on this crop remain poorly explored under salt stress conditions. Therefore, further investigations are needed to elucidate, in greater detail, the biochemical, nutritional, and antioxidant mechanisms through which selenium reduces or mitigates the impacts of irrigation water salinity.

## Figures and Tables

**Table 1:** Water consumption of okra plants during the experimental period.

ECw (dS m^−1^)	Average Water Consumption (L)	Average Water Consumption * (mm)
0.4	63.25	838.01
1.3	57.5	761.82
2.2	41.5	549.84
3.1	33.75	447.16

* Water depth calculated taking into account the area of the pot (0.0755 m^2^).

**Table 2:** Volume of selenium spray solution applied per plant at 21, 35, and 50 days after sowing (DAS).

Selenium Application Dates	Q1 (mL)	Q2 (mL)	Q3 (mL)	Q4 (mL)
14 April 2025	0	41.67	25	19.17
28 April 2025	0	154.17	158.33	165.83
15 March 2025	0	150	166.67	125

Q1, Q2, Q3, and Q4 correspond to selenium concentrations of 0, 5, 10, and 15 mg L^−1^, respectively.

## Data Availability

The original contributions presented in this study are included in the article/Supplementary Materials. Further inquiries can be directed to the corresponding author.

## Supplementary Materials

The following supporting information can be downloaded at: https://www.mdpi.com/article/10.3390/plants15010021/s1, Table S1. Summary of the analysis of variance for relative water content (RWC) and electrolyte leakage (EL) in okra cultivated under different irrigation water salinity levels and selenium concentrations, at 65 days after sowing. Table S2. Summary of the analysis of variance for intercellular CO_2_ concentration (*Ci*), stomatal conductance (*gs*), transpiration (*E*), CO_2_ assimilation rate (*A*), instantaneous water-use efficiency (*WUEi*), and instantaneous carboxylation efficiency (*CEi*) in okra cultivated under different irrigation water salinity levels and selenium concentrations, at 65 days after sowing. Table S3. Summary of the analysis of variance for chlorophyll *a* (Chl *a*), chlorophyll *b* (Chl *b*), total chlorophyll (Chl total), and carotenoid (Car) contents in okra cultivated under different irrigation water salinity levels and selenium concentrations, at 65 days after sowing. Table S4. Summary of the analysis of variance for initial fluorescence (F_o_), maximum fluorescence (Fm), variable fluorescence (Fv), and the quantum efficiency of photosystem II (Fv/Fm) in okra cultivated under different irrigation water salinity levels and selenium concentrations, at 65 days after sowing. Table S5. Summary of the analysis of variance for plant height (PH), stem diameter (SD), number of leaves (NL), and leaf area (LA) in okra cultivated under different irrigation water salinity levels and selenium concentrations, at 65 days after sowing. Table S6. Summary of the analysis of variance for leaf dry mass (LDM), stem dry mass (SDM), root dry mass (RDM), total aboveground biomass (TAB), and root-to-shoot ratio (RSR) in okra cultivated under different irrigation water salinity levels and selenium concentrations, at 94 days after sowing. Table S7. Summary of the analysis of variance for number of fruits per plant (NFP), mean fruit weight (MFW), mean fruit diameter (MFD), and total fruit production per plant (FPP) in okra cultivated under different irrigation water salinity levels and selenium concentrations, from 60 to 94 days after sowing. Table S8. Summary of the analysis of variance for water-use efficiency (WUE) and water consumption (WC) in okra plants cultivated under different irrigation water salinity levels and selenium concentrations, from 60 to 94 days after sowing.
