# Supplementary material for: Foliar Application of Selenium in Mitigating Salinity Stress on the Physiology, Growth, and Yield of Okra
Source: Plants (Basel). 2025 Dec 20;15(1):21. doi: 10.3390/plants15010021 (PMC12787630; doi:10.3390/plants15010021)
Supplement: Supplementary file 1 [file plants-15-00021-s001.zip › plants-4041709-supplementary.pdf]

Table S1. Summary of the analysis of variance for relative water content (RWC) and electrolyte leakage (EL) in okra cultivated under different irrigation water salinity levels and selenium concentrations, at 65 days after sowing.

**Source of variation**	**DF**	**Mean squares**	
		**RWC**	**EL**
Electrical conductivity (ECw)	3	61.15***	295.43***
Linear regression	1	176.42***	819.13***
Quadratic regression	1	5.54^{ns}	64.09*
Selenium concentrations (Se)	3	21.66*	10.05^{ns}
Linear regression	1	42.16*	6.98^{ns}
Quadratic regression	1	0.83^{ns}	17.56^{ns}
Interaction (ECw × Se)	9	17.11*	40.18$^{=}$
Blocks	2	17.31^{ns}	6.89^{ns}
Residue	30	6.97	9.88
CV (%)		3.27	7.35

DF – Degrees of freedom; CV – Coefficient of variation; *, **, ***, ns – Significant at $p \leq 0.05$, $p \leq 0.01$, $p \leq 0.001$, and not significant by F test, respectively.

Table S2. Summary of the analysis of variance for intercellular CO_2 concentration (*Ci*), stomatal conductance (*gs*), transpiration (*E*), CO_2 assimilation rate (*A*), instantaneous water-use efficiency (*WUEi*), and instantaneous carboxylation efficiency (*CEi*) in okra cultivated under different irrigation water salinity levels and selenium concentrations, at 65 days after sowing.

**Source of variation**	**DF**	**Mean squares**					
		gs	***E***	***Ci***	***A***	***WUEi***	***CEi***
Electrical conductivity (ECw)	3	0.0019***	0.378*	533.743^{ns}	21.1467**	2.3476**	0.000905***
Linear regression	1	0.0051***	1.0143**	676.7042^{ns}	59.7253**	2.5060*	0.0027***
Quadratic regression	1	0.0001^{ns}	0.0657^{ns}	841.6875^{ns}	0.8144^{ns}	0.5897^{ns}	0.000038^{ns}
Selenium concentrations (Se)	3	0.0035***	0.2233^{ns}	551.5208^{ns}	6.2019**	1.2195^{ns}	0.000074**
Linear regression	1	0.007***	0.3116^{ns}	418.7042^{ns}	2.6052^{ns}	0.8902^{ns}	0.000021^{ns}
Quadratic regression	1	0.0029***	0.3458^{ns}	1036.0208^{ns}	14.1429**	0.6175^{ns}	0.000189***
Interaction (ECw × Se)	9	0.0005*	0.2623*	409.6134^{ns}	3.6701**	3.3387**	0.000114***
Blocks	2	0.00066^{ns}	0.4914*	1514.5833^{ns}	1.1457^{ns}	0.2458^{ns}	0.000001^{ns}
Residue	30	0.0068	0.1045	874.3167	0.7991	0.4306	0.000012
CV (%)		8.76	16.98	12.39	6.60	9.00	6.15

DF – Degrees of freedom; CV – Coefficient of variation; *, **, ***, ns – Significant at $p \leq 0.05$, $p \leq 0.01$, $p \leq 0.001$, and not significant by F test, respectively.

Table S3. Summary of the analysis of variance for chlorophyll *a* (Chl *a*), chlorophyll *b* (Chl *b*), total chlorophyll (Chl total), and carotenoid (Car) contents in okra cultivated under different irrigation water salinity levels and selenium concentrations, at 65 days after sowing.

**Source of variation**	**DF**	**Mean squares**			
		**Chl *a***	**Chl *b***	**Chl t**	**Car**
Electrical conductivity (ECw)	3	120453.07***	3917.47**	167577.16***	14367.98***
Linear regression	1	260873.38***	9511.39**	370009.49***	33539.13***

Quadratic regression	1	69519.99***	1619.97^{ns}	92364.52***	8041.70**
Selenium concentrations (Se)	3	68926.59***	2484.23^{ns}	94317.93***	12253.33***
Linear regression	1	155590.97***	2765.06^{ns}	199839.42***	17186.36***
Quadratic regression	1	14632.36^{ns}	21.29^{ns}	15769.95^{ns}	6680.64**
Interaction (ECw × Se)	9	30992.05***	970.68^{ns}	37273.08***	2712.65**
Blocks	2	302.22^{ns}	215.18^{ns}	531.42^{ns}	225.27^{ns}
Residue	30	3986.85	816.95	5483.19	871.23
CV (%)		3.03	5.72	2.87	4.11

DF – Degrees of freedom; CV – Coefficient of variation; *, **, ***, ns – Significant at p ≤ 0.05, p ≤ 0.01, p ≤ 0.001, and not significant by F test, respectively.

Table S4. Summary of the analysis of variance for initial fluorescence (F_o), maximum fluorescence (Fm), variable fluorescence (Fv), and the quantum efficiency of photosystem II (Fv/Fm) in okra cultivated under different irrigation water salinity levels and selenium concentrations, at 65 days after sowing.

Source of variation	DF	Mean squares			
		F_o	Fm	Fv	Fv/Fm
Electrical conductivity (ECw)	3	310.08**	2864.58*	4282.97**	0.000651***
Linear regression	1	567.34**	7992.60**	12818.82***	0.001801***
Quadratic regression	1	315.19*	513.52^{ns}	24.08^{ns}	0.000128^{ns}
Selenium concentrations (Se)	3	139.41^{ns}	428.41^{ns}	590.08^{ns}	0.000147^{ns}
Linear regression	1	288.20*	196.20^{ns}	8.82^{ns}	0.000173^{ns}
Quadratic regression	1	58.52^{ns}	1.02^{ns}	44.08^{ns}	0.000045^{ns}
Interaction (ECw × Se)	9	232.53**	848.08^{ns}	1167.12^{ns}	0.000262**
Blocks	2	15.08^{ns}	1568.69^{ns}	1307.52^{ns}	0.000032^{ns}
Residue	30	58.68	727.31	651.65	0.00006
CV (%)		3.23	2.57	3.15	1.00

DF – Degrees of freedom; CV – Coefficient of variation; *, **, ***, ns – Significant at p ≤ 0.05, p ≤ 0.01, p ≤ 0.001, and not significant by F test, respectively.

Table S5. Summary of the analysis of variance for plant height (PH), stem diameter (SD), number of leaves (NL), and leaf area (LA) in okra cultivated under different irrigation water salinity levels and selenium concentrations, at 65 days after sowing.

Source of variation	GL	Mean squares			
		PH	SD	NL	LA
Electrical conductivity (ECw)	3	378.02***	7.52***	67,25***	3528682.89***
Linear regression	1	1122.34***	19.88***	106,67^{ns}	9743656.03***
Quadratic regression	1	6.02^{ns}	2.61*	1,33^{ns}	4325.18^{ns}
Selenium concentrations (Se)	3	17.08^{ns}	5.82***	53.36***	436088.67^{ns}
Linear regression	1	47.07*	11.92***	32.27**	1119550.82*
Quadratic regression	1	3.52^{ns}	0.16^{ns}	126.75***	95564.59^{ns}
Interaction (ECw × Se)	9	16.13*	2.03**	22.53***	582770.33*
Blocks	2	23.25^{ns}	0.45^{ns}	3.39^{ns}	72094.10^{ns}
Residue	30	7.16	0.65	2.95	190515.27
CV (%)		5.20	5.63	10.88	9.12

DF – Degrees of freedom; CV – Coefficient of variation; *, **, ***, ns – Significant at p ≤ 0.05, p ≤ 0.01, p ≤ 0.001, and not significant by F test, respectively.

Table S6. Summary of the analysis of variance for leaf dry mass (LDM), stem dry mass (SDM), root dry mass (RDM), total aboveground biomass (TAB), and root-to-shoot ratio (RSR) in okra cultivated under different irrigation water salinity levels and selenium concentrations, at 94 days after sowing.

Source of variation	DF	Mean squares				
		DLP	DSP	DRP	TAP	RSR
Electrical conductivity (ECw)	3	124.91***	69.29***	209.93***	313.92***	0.04**
Linear regression	1	251.12***	190.04***	491.06***	878.08***	0.0008^{ns}
Quadratic regression	1	105.39***	17.14^{ns}	8.85^{ns}	37.52*	0.00007^{ns}
Selenium concentrations (Se)	3	12.15^{ns}	30.95**	331.13***	36.74*	0.13***
Linear regression	1	25.41*	1.99^{ns}	8.88^{ns}	13.18^{ns}	0.014^{ns}
Quadratic regression	1	11.02^{ns}	0.06^{ns}	669.61***	9.48^{ns}	0.34***
Interaction (ECw × Se)	9	23.64***	15.40*	195.71***	29.36**	0.12***
Blocks	2	6.92^{ns}	5.34^{ns}	58.53**	24.24^{ns}	0.02^{ns}
Residue	30	4.78	5.25	10.02	8.25	0.007
CV (%)		8.35	10.80	10.14	6.06	12.81

DF – Degrees of freedom; CV – Coefficient of variation; *, **, ***, ns – Significant at $p \leq 0.05$, $p \leq 0.01$, $p \leq 0.001$, and not significant by F test, respectively.

Table S7. Summary of the analysis of variance for number of fruits per plant (NFP), mean fruit weight (MFW), mean fruit diameter (MFD), and total fruit production per plant (FPP) in okra cultivated under different irrigation water salinity levels and selenium concentrations, from 60 to 94 days after sowing.

Source of variation	DF	Mean squares			
		NFP	MFW	MFD	FPP
Electrical conductivity (ECw)	3	27.81***	39.27**	0.65^{ns}	12518.25***
Linear regression	1	1.67^{ns}	83.14***	0.002^{ns}	6505.52***
Quadratic regression	1	80.08***	28.39*	0.008^{ns}	25986.54***
Selenium concentrations (Se)	3	3.58**	35.89**	1.24*	3266.35***
Linear regression	1	6.67**	33.22*	0.03^{ns}	7291.60***
Quadratic regression	1	0.33^{ns}	73.29**	2.52**	2428.92**
Interaction (ECw × Se)	9	8.71***	19.15**	0.97**	3996.02***
Blocks	2	0.39^{ns}	2.44^{ns}	0.18^{ns}	201.30^{ns}
Residue	30	0.68	5.04	0.28	297.89
CV (%)		11.75	10.18	3.28	11.25

DF – Degrees of freedom; CV – Coefficient of variation; *, **, ***, ns – Significant at $p \leq 0.05$, $p \leq 0.01$, $p \leq 0.001$, and not significant by F test, respectively.

Table S8. Summary of the analysis of variance for water-use efficiency (WUE) and water consumption (WC) in okra plants cultivated under different irrigation water salinity levels and selenium concentrations, from 60 to 94 days after sowing.

Source of variation	DF	Mean squares
		WUE
Electrical conductivity (ECw)	3	0.033***
Linear regression	1	0.063***
Quadratic regression	1	0.035***
Selenium concentrations (Se)	3	0.005**
Linear regression	1	0.01***

Quadratic regression	1	0.004*
Interaction (ECw × Se)	9	0.010***
Blocks	2	0.0006^{ns}
Residue	30	0.0008
CV (%)		11.77
Source of variation	GL	Mean squares
		WC
Electrical conductivity (ECw)	3	1.19**
Linear regression	1	1150.16***
Quadratic regression	1	2106.47***
Blocks	2	1.41^{ns}
Residue	40	1.59
CV (%)		0.00

DF – Degrees of freedom; CV – Coefficient of variation; *, **, ***, ns – Significant at $p \leq 0.05$, $p \leq 0.01$, $p \leq 0.001$, and not significant by F test, respectively.
